# Inhibitory effects of transcription factor Ikaros on the expression of liver cancer stem cell marker CD133 in hepatocellular carcinoma

**DOI:** 10.18632/oncotarget.2524

**Published:** 2014-09-25

**Authors:** Lin Zhang, Hong Li, Chao Ge, Meng Li, Fang-yu Zhao, He-lei Hou, Miao-xin Zhu, Hua Tian, Li-xing Zhang, Tao-yang Chen, Guo-ping Jiang, Hai-yang Xie, Ying Cui, Ming Yao, Jin-jun Li

**Affiliations:** ^1^ State Key Laboratory of Oncogenes and Related Genes, Shanghai Cancer Institute, Renji Hospital, Shanghai Jiaotong University School of Medicine, Shanghai, China; ^2^ Qi Dong Liver Cancer Institute, Qi Dong, China; ^3^ Department of General Surgery, the First Affiliated Hospital, School of Medicine, Zhejiang University, Hangzhou, China; ^4^ Cancer Institute of Guangxi, Nanning, China

**Keywords:** Ikaros, CD133, hepatocellular carcinoma, cancer stem cells

## Abstract

CD133 is a cellular surface glycoprotein that has been reported as a marker for the enrichment of cancer stem cells (CSCs). However, the regulatory mechanism of CD133 remains unknown. CSCs have been proposed to contribute to radioresistance and multi-drug resistance. The elucidation of key regulators of CD133 and CSCs is critical for the development of CSC-targeted therapy. In this study, we showed that Ikarosinhibited the expression of CD133 via direct binding to the CD133 P1 promoter and repressed the tumorigenic and self-renewal capacity of CD133^+^ cancer stem-like cells in hepatocellular carcinoma (HCC). We found that Ikaros interacted with CtBP as a transcription repressor complex, which inhibited CD133 expression in HCC. We also demonstrated that Ikaros expression was up-regulated by ETS1 which activity was regulated by MAPKs pathway. Furthermore, decreased expression of Ikaroswas significantly associated with poor survival in HCC patients. Overall, our study identifies that Ikaros plays a role as a transcription repressor in HCC and is a new reactivated therapeutic target for the treatment of HCC. Meanwhile, our findings provide evidence that Ikaros could be an attractive inhibitor of the target gene CD133, which reactivates anticancer mechanisms in targeted CSC therapy.

## INTRODUCTION

HCC is one of the most common malignancies worldwide [1]. Currently, there is no effective systemic chemotherapy for patients with advanced HCC because HCC is relatively chemotherapy-resistant and exhibits high rates of recurrence. Cancer stem cells (CSCs) are generally considered to be key contributors to tumor initiation, maintenance, chemoresistance, radioresistance, recurrence, and metastasis. CD133^+^ CSCs from lung cancer are in enrichment after cisplatin treatment and have stable cisplatin-resistant phenotype *in vitro* [2]. Thus, the efficient identification and isolation of CSCs from cancer cell lines or tumor tissues is necessary and helpful for the development of anti-cancer strategies targeting CSCs. Multiple of cell surface markers such as CD13, CD44, DLK1, ALDH1, CD133 have been used to isolate and identify CSCs in solid tumors [3-10], in which CD133 has been identified as a marker for enrichment of CSCs in HCC. Compared with CD133^−^ cells, CD133^+^ cells exhibit significantly higher proliferation potential as well as a greater capacity for colony formation and tumor initiation *in vivo* [5-10]. CD133^+^ cells are typically reactivated in HCC, and the expression of CD133 increases in higher-stage tumors. This enhancement of CD133 expression typically indicates poor patient prognosis [11]. However, little is known regarding the characteristics and molecular regulation of CD133 and CD133^+^ cancer stem-like cells in HCC.

Ikaros is a member of the Kruppel family of zinc finger DNA-binding protein [12], which is the crucial regulator of normal hematopoiesis, and has been identified as a tumor suppressor associated with the development of leukemia [13, 14, 15]. Loss of Ikaros function is thought to be essential to the development of lymphoid leukemia, including adult B-cell acute lymphoblastic leukemia (B-ALL), acute myeloid leukemia and infant T-cell ALL [13, 16, 17]. Ikaros mutation or polymorphism is a typical genetic feature in human B-ALL [18]. In general, Ikaros binds to promoter regions and regulates target gene expression by recruiting promoter regions into pericentromeric heterochromatin. As a result, its target genes are suppressed via chromatin remodeling. The expression of *Hes1* and *Deltex* are repressed by the direct binding of Ikaros to their promoters [19, 20]. Ikaros also plays important role in the development of nervous system, and the cerebral cortex and hypothalamic–pituitary somatotroph development [21, 22]. The essential function of Ikaros in leukemia is obvious; however, the role of Ikaros in solid tumors remains unclear, particularly in hepatocellular carcinoma (HCC).

In this study, we showed that Ikaros interacted with the transcription repressor CtBP as a complex and inhibited CD133 expression via direct binding to the CD133 P1 promoter in HCC. Moreover, biological activation of CD133^+^ cancer stem-like cells was regulated by Ikaros in HCC. We confirmed that ETS1 up-regulated the expression of Ikaros. In addition, patients with higher Ikaros expression had longer survival. Thus, our results suggest that reactivation of Ikaros could be a novel strategy for treatment of HCC.

## RESULTS

### P1 promoter is the main 5′-UTR pattern of CD133 in HCC

**Figure 1:**
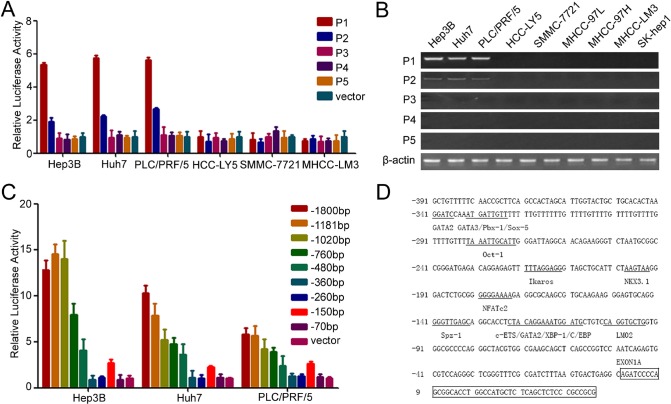
P1 promoter is the key promoter of CD133 in HCC (A) The promoter activity of P1, P2, P3, P4, and P5 in HCC cells. (B) RT-PCR analysis of CD133 expression pattern for 5′-UTR containing exons 1A (P1), 1B (P2), 1C (P3), 1D (P4), and 1E (P5) in HCC cells. (C) The promoter activity of the deleted P1 promoter in Hep3B, Huh7, and PLC/PRF/5 cells. (D) Analysis of CD133 P1 promoter. Transcription factors were predicted using TFSEARCH (http://mbs.cbrc.jp/research/db/TFSEARCH.html).

In our previous study, we showed that CD133 was used to isolate and identify CSCs in HCC and CD133^+^ HCC cells exhibited the biological characteristics of cancer stem cell in HCC [6, 7]. To understand the regulatory mechanism of CD133 expression, we examined the CD133 promoter. Five alternative promoters (P1, P2, P3, P4, and P5) were identified in the 5′-UTR of CD133 (Supplementary Figure S1A) [23]. Reporter assays revealed that the P1 promoter exhibited the highest activity of the five pattern promoters of CD133 in Hep3B, Huh7 and PLC/PRF5 (Figure 1A), which have higher expressions of CD133 mRNA and protein (Supplementary Figure S1B and C), and RT-PCR further confirmed that the P1 promoter was the principal promoter of CD133 (Figure 1B).

We successively deleted the sequences of P1 promoter and investigated the activity of these promoters. The promoter activity was increased when −260/−150-bp sequences were deleted (Figure 1C). We concluded that this region might contain transcription repressor binding sites. We further analyzed the transcription factor binding sites in the P1 promoter and multiples of transcription factors sites were found in this region (Figure 1D). Some of these transcription factors were able to down-regulate the expression of CD133, such as c-ETS1, Ikaros and Nkx3.1 (Supplementary Figure S1D). Taken together, these results showed that the P1 promoter was the key promoter of CD133 in HCC.

### Ikaros inhibits CD133 expression by directly binding to the CD133 promoter

**Figure 2:**
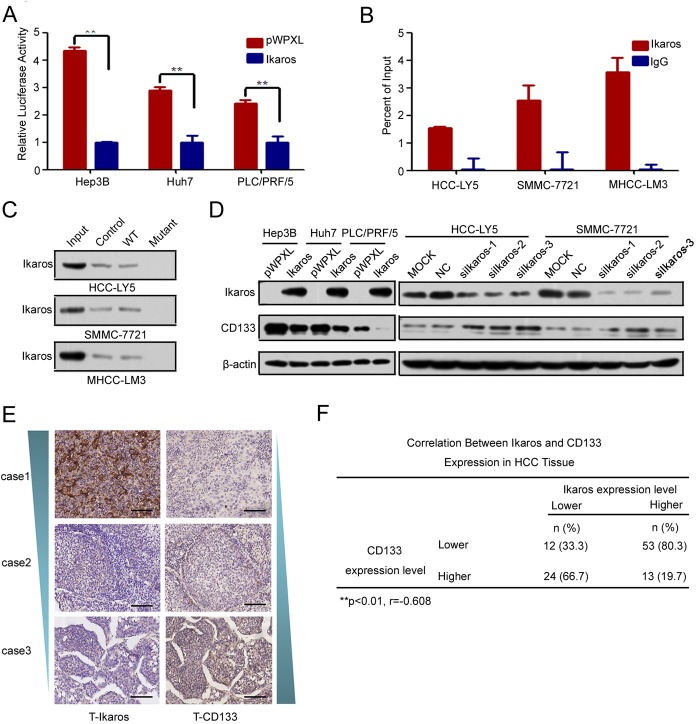
Ikaros represses CD133 expression in HCC (A) The promoter activity of P1 after the transfection of Ikaros into Hep3B, Huh7, and PLC/PRF/5 (PLC) cells. **, p<0.01. (B) Binding of Ikaros on the CD133 gene promoter was assayed by chromatin immunoprecipitation (ChIP). ChIP was performed using antibodies to Ikaros in HCC-LY5, SMMC-7721, and MHCC-LM3 cells. A negative control with irrelevant antibody (IgG) was included for comparison. qPCR was performed on the bound fractions. (C) DNA pull-down assay to detect Ikaros binding to the P1 promoter using either wild-type (WT) or Ikaros mutant oligonucleotide sequences from the CD133 promoter in HCC-LY5, SMMC-7721, and MHCC-LM3 cells. (D) Immunoblot analysis of CD133 expression when Ikaros was overexpressed or silenced. (E) IHC analysis of Ikaros and CD133 in HCC samples. Representative images are shown. Scale bar, 100 μm. (F) The analysis of the correlation between Ikaros and CD133 in HCC tissue. **, p<0.01; r=-0.608; n=102. Data represent mean ± SD of three independent experiments with technical triplicates for each, and statistical analysis was performed using Student's *t* test.

One Ikaros binding site was observed at −212/−221-bp region in the CD133 P1 promoter. Ikaros is a crucial regular in the differentiation of hematopoietic stem cells and has been identified as a tumor suppressor associated with the development of leukemia [13, 14, 24, 25]. To identify whether Ikaros also played an important role in CD133 expression, we examined the P1 promoter activity when Ikaros was overexpressed. Report assays showed that the P1 promoter activity was inhibited when Ikaros was enforced to express in HCC cells (Figure 2A). ChIP assays and DNA pull-down assays confirmed that Ikaros directly bound to the CD133 P1 promoter in HCC cells (Figure 2B and C). However, CD133 promoter activity was unchanged in response to Ikaros overexpression when the Ikaros binding site in the CD133 promoter was mutated (Supplementary Figure S2A). The 157 phenylalanine (F), 162 arginine (R), and 184 arginine (R) have been confirmed to be necessary for Ikaros DNA binding activity and subcellular localization [26]. Therefore, we constructed three mutants in which these three critical amino acids were mutated to alanine (A). Report assays showed that P1 promoter activity had no change when these mutant Ikaros were overexpressed (Supplementary Figure S2B).

To further determine whether Ikaros could down-regulate CD133 expression, CD133 expression was detected after ectopic overexpression or knockdown of Ikaros. Overexpressed Ikaros decreased the expression of CD133, while silenced Ikaros increased CD133 expression (Figure 2D). The mRNA and protein levels of Ikaros and CD133 were negatively correlated in nine HCC cell lines using qPCR and western blot (Supplementary Figure S1B and C). To better understand the correlation between Ikaros and CD133, immunohistochemical staining (IHC) analysis was performed in 102 human primary HCC samples. IHC analysis showed that Ikaros protein levels inversely correlated with CD133 expression in HCC tissues (Figure 2E and F). Together, these results suggested that Ikaros inhibited the expression of CD133 via direct binding to the CD133 P1 promoter in HCC.

### CtBP could interact with Ikaros and regulate CD133 expression in HCC cells

**Figure 3:**
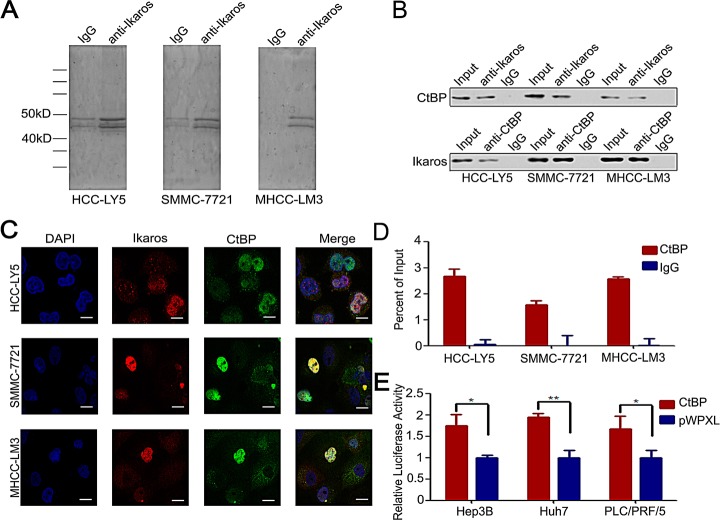
CtBP interacts with Ikaros as a transcription repressor complex (A) Co-immunoprecipitation assay to detect Ikaros interaction proteins in HCC-LY5, SMMC-7721, and MHCC-LM3 cells. (B) Immunoblot analysis of co-immunoprecipitated mixture. (C) Confocal imaging analysis of CtBP and Ikaros colocalization in the nucleus. Red indicates Ikaros; green indicates CtBP; and blue indicates DAPI. Scale bar, 10 μm. (D) Binding of CtBP on the CD133 gene promoter was assayed by ChIP, which was performed using antibodies to CtBP in HCC-LY5, SMMC-7721, and MHCC-LM3 cells. A negative control with irrelevant antibody (IgG) was included for comparison. qPCR was performed on the bound fractions. (E) The promoter activity of P1 after the transfection of CtBP into Hep3B, Huh7, and PLC/PRF/5 cells. **, p<0.01; *, p<0.05. Data represent mean ± SD of three independent experiments with technical triplicates for each, and statistical analysis was performed using Student's *t* test.

Usually, Ikaros interacts with co-repressors as complexes to regulate target genes. The co-immunoprecipitation (Co-IP) assay was performed to identify the co-repressors of Ikaros in HCC cells. Approximately, 40-50 kD proteins were precipitated from the three HCC cell lines (Figure 3A). We suspected that CtBP, with a molecular weight of approximately 48 kD, interacted with Ikaros as a co-repressor [27]. To determine whether Ikaros interacts with CtBP in HCC, we analyzed precipitated samples using western blot. CtBP was identified in anti-Ikaros precipitated samples and Ikaros was also detected in anti-CtBP precipitated fractions (Figure 3B). Next, we investigated the subcellular location of Ikaros and CtBP using immunofluorescence staining. Results demonstrated that these proteins were co-localized in HCC cells (Figure 3C). ChIP assays showed CtBP was able to bind to CD133 P1 promoter, which further confirmed the interaction between CtBP and Ikaros (Figure 3D). Uniformly, the CD133 promoter activity was inhibited by overexpression of CtBP (Figure 3E). Taken together, these findings confirmed that CtBP interacted with Ikaros and regulated CD133 expression in HCC.

### ETS1 up-regulates Ikaros expression via the MAPKs pathway

**Figure 4:**
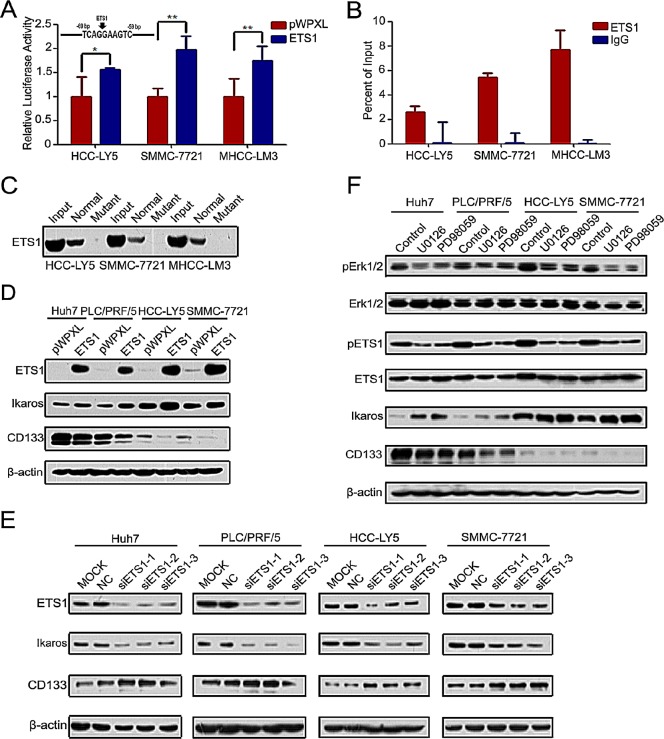
MAPKs/ETS1 pathway regulates the expression of Ikaros and CD133 (A) The promoter activity of Ikaros responding to ETS1 overexpressing in HCC-Y5, SMMC-7721, and MHCC-LM3 cells. **, p<0.01; *, p<0.05. The Ikaros promoter was analyzed using TFSEARCH (http://mbs.cbrc.jp/research/db/TFSEARCH.html). (B) Binding of ETS1 on the Ikaros gene promoter was assayed by ChIP which was performed using antibodies to ETS1 in HCC-LY5, SMMC-7721, and MHCC-LM3 cells. A negative control with irrelevant antibody (IgG) was included for comparison. qPCR was performed on the input and bound fractions. (C) DNA pull-down assay to detect ETS1 binding to the Ikaros promoter in HCC-LY5, SMMC-7721, and MHCC-LM3 cells. (D and E) Immunoblot analysis of Ikaros and CD133 expression when ETS1 overexpression or knockdown. (F) Immunoblot analysis of the expression of Erk1/2, pErk1/2, ETS1, pETS1, Ikaros, and CD133 when specific MAPKs inhibitors (U0126, PD98059) were added to Huh7, PLC/PRF/5, HCC-LY5, and SMMC-7721 cells. Data represent mean ± SD of three independent experiments with technical triplicates for each, and statistical analysis was performed using Student's *t* test.

To detect which pathway participates in the regulation of Ikaros and CD133 expression, we analyzed the Ikaros promoter. One binding site for the transcription factor ETS1 was found in the Ikaros promoter at the −69/−59-bp region (Figure 4A). Reporter assays showed that Ikaros promoter activity was significantly enhanced in response to ETS1 overexpression (Figure 4A). Ohnishi *et al* showed that the ETS family transcription factors regulate CD133 via direct binding of CD133 to the P5 promoter in colon cancer cells [28]. One ETS1 binding site was also predicted in the CD133 P1 promoter. However, ChIP assays revealed that ETS1 did not bind to this site (Supplementary Figure S3A).

ChIP assay and DNA pull-down assay further confirmed that ETS1 directly bound to the Ikaros promoter (Figure 4B and C). When ETS1 was silenced, we did not detect the binding between ETS1 and Ikaros promoter (Supplementary Figure S3B). Ikaros expression was upregulated by overexpressing ETS1 and down-regulated by ETS1 knockdown, while CD133 expression exhibited the opposite changes compared with Ikaros (Figure 4D and E). ETS1 activity was inhibited via phosphorylated by Erk1/2 [29, 30]. In addition, specific inhibitors PD98059 and U0126 were utilized to block Erk1/2 activity. The expression of phosphorylated Erk1/2 and ETS1, Ikaros, and CD133 also correspondingly changed after treatment with these inhibitors (Figure 4F). Taken together, these results indicated that the MAPKs/ETS1 pathway regulates Ikaros and CD133 expression in HCC.

### Ikaros inhibits the tumorigenic and self-renewal capacity of CD133^+^ HCC cells

**Figure 5:**
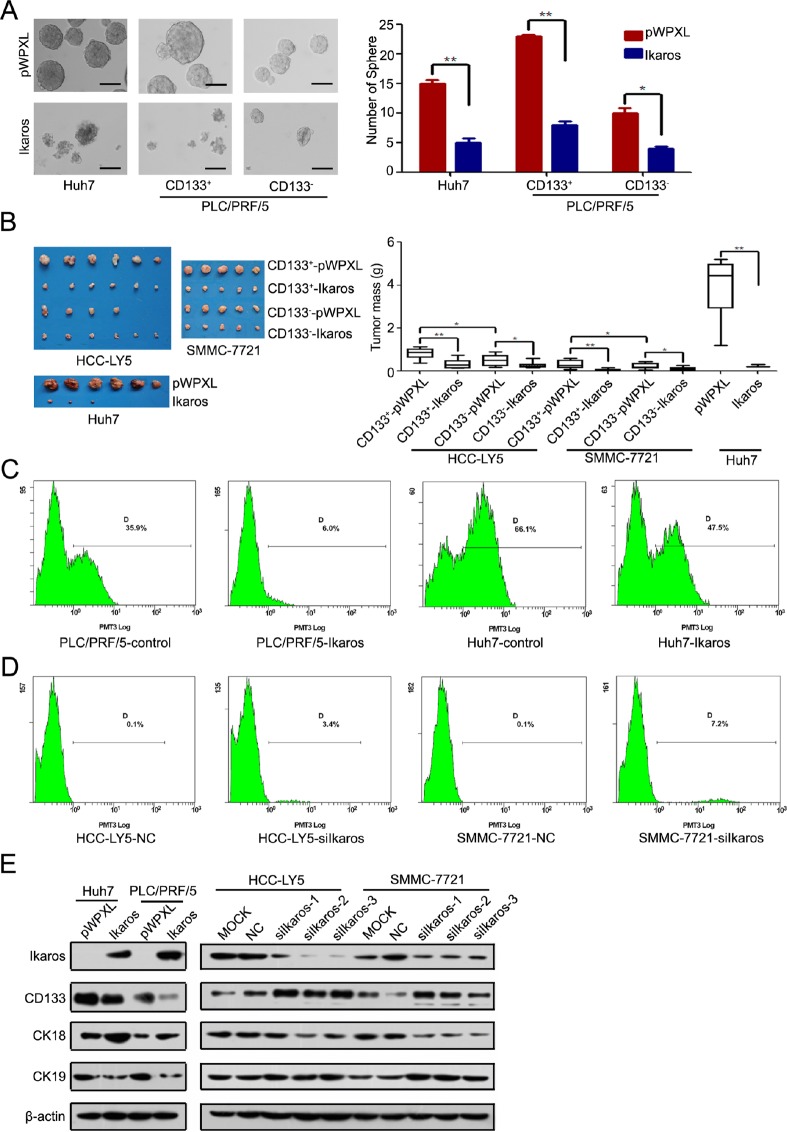
Ikaros inhibits the tumor growth and self-renewal of CD133 HCC cells (A) A representative image of a CSC sphere in self-renewal assays (left panel). The mean values of Huh7, CD133^+/−^ PLC/PRF/5 cells sphere from three independent experiments are shown in the right panel. (Scale bar, 100 μm) (B) The size of tumors from NOD/SCTD mice injected with Ikaros-overexpressing or control cells (left panel). The weight of tumors from NOD/SCTD mice injected with Ikaros-overexpressing or control cells in the right panels. **, p<0.01; *, p<0.05. (C) Flow cytometric analysis of the percentage of CD133^+^ cells when Ikaros was overexpressed in Huh7 and PLC/PRF/5 cells. (D) Flow cytometric analysis of the percentage of CD133^+^ cells when Ikaros was knockdown in HCC-LY5 and SMMC-7721 cells. (E) Immunoblot analysis of expression of CD133, CK18, and CK19 when Ikaros was enforced to express or silenced. Data represent mean ± SD of three independent experiments with technical triplicates for each, and statistical analysis was performed using Student's *t* test.

Next, we determined the function of Ikaros in HCC. Cell-proliferation assay revealed that forced Ikaros expression remarkably inhibited the cell growth of HCC cells *in vitro* (Supplementary Figure S4A). We found that Ikaros had more effect to HCC cells with high-level expression of CD133. Colony-formation assay using sorted CD133^+/−^ cells from HCC-LY5, SMMC-7721 and MHCC-LM3 cells also showed that the suppression of growth by Ikaros overexpression was more effective in CD133^+^ cells than CD133^−^ cells (Supplementary Figure S4B). The self-renewal capacity of Huh7 and CD133^+^ PLC/PRF/5 cells was significantly inhibited by transfection with Ikaros (Figure 5A). When HCC cells transfected with Ikaros were treated with Cisplatin, the IC50 value was decreased. These cells were also more susceptible to Doxorubicin and Sorafenib (Supplementary Figure S4C). Next, CD133^+^ or CD133^−^ cells transfected with Ikaros or control from SMMC-7721 or HCC-LY5 were subcutaneously injected into non-obese diabetic/severe combined immunodeficiency (NOD/SCID) mice. Ikaros significantly inhibited the tumorigenicity of CD133+ cells (Figure 5B). Western blot showed that the expression of CD133 from xenograft mouse models was inhibited by Ikaros *in vivo* (Supplementary Figure S4D).

Ikaros not only repressed CD133 expression, but also decreased the proportion of CD133^+^ HCC cells upon forced Ikaros expression (Huh7 cells for around 28.1%; PLC/PRF/5 cells for around 83.2%) (Figure 5C). In addition, the proportion of CD133^+^ HCC cells were increased when Ikaros was silenced (HCC-LY5 cells for approximately 34 times; SMMC-7721 cells for approximately 72 times) (Figure 5D). We further examined differentiated molecular marker Cytokeratin 18 (CK18) and Cytokeratin 19 (CK19) in Ikaros-transfected HCC cells. CK18 and CK 19 were increased and decreased, respectively, when Ikaros was overexpressed. Moreover, CK18 and CK19 had opposite changes after Ikaros was knocked down (Figure 5E). These data confirmed that Ikaros could efficiently inhibit tumorigenicity and the self-renewal capacity of CD133^+^ CSCs in HCC.

### High Ikaros levels indicate longer survival in HCC patients

**Figure 6:**
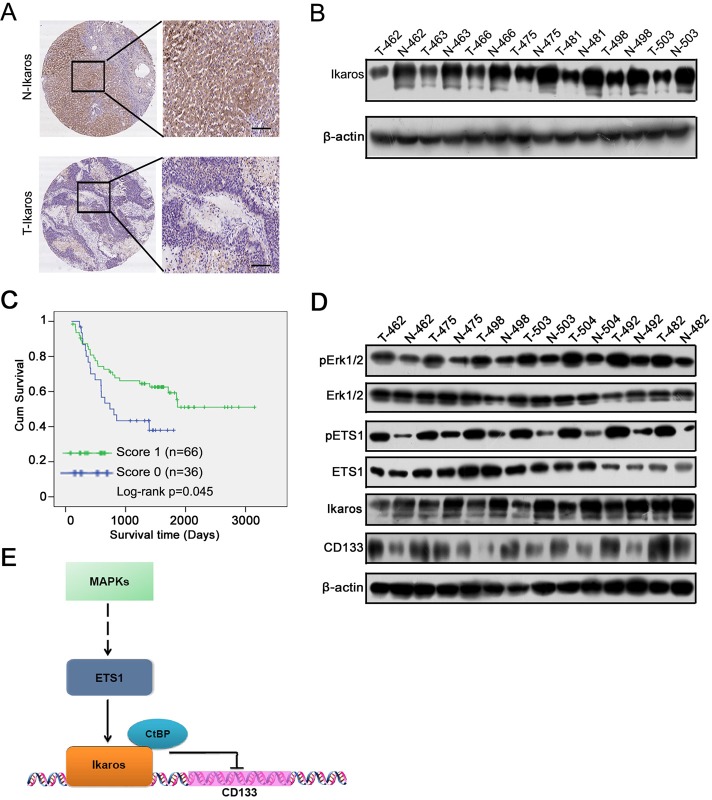
The clinical significance of Ikaros in HCC (A) The IHC analysis of Ikaros in HCC samples. Scale bar, 100 μm. (B) Immunoblot analysis of Ikaros expression in HCC samples. (C) The overall survival analysis of HCC patients with Ikaros expression. *, p<0.05; n=102; Log-rank p=0.045. (D) Immunoblot analysis of the expression of Erk1/2, ETS1, Ikaros, and CD133 in HCC samples. (E) The function model of Ikaros in HCC. MAPK pathway regulates the activity of ETS1 via phosphorylation of ETS1. ETS1 is able to up-regulate Ikaros expression, and Ikaros interacting with CtBP inhibits CD133 expression via directly binding to CD133 P1 promoter.

We analyzed Ikaros expression in 102 pairs of human primary HCC and matched noncancerous liver specimens using IHC. The IHC analysis demonstrated that Ikaros expression was at low levels in HCC samples (Figure 6A), and 17.8% (18/102) of human primary HCC specimens with Ikaros protein staining was higher than paired nontumorous liver specimens. Western blot showed that the protein levels of Ikaros were down-regulated in primary HCC tissues (Figure 6B).

According to the IHC results, the expression intensity of Ikaros protein was scored as 0, 1 for weak and strong immunostaining, respectively. The results showed that Ikaros expression was negatively associated with histological grade of HCC and tumor size (Table 1). However, there was no correlation between Ikaros expression and other clinicopathological indexes, including age, gender, serum alpha-fetoprotein (AFP), intrahepatic metastasis, hepatitis B surface antigen (HBsAg) levels, and with or without cirrhosis (Table 1). Further, overall survival analysis revealed that the high expression of Ikaros was closely associated with good outcomes in HCC patients (Figure 6C).

**Table 1:** Relationship between Ikaros expression and clinicopathological features in HCC tissues

Clinicopathological Features		Ikaros Expression (Cancer)		
		Score 0	Score 1	
	Case	Case (%)	Case (%)	p Value
Age (years)				
<60	76	28 (77.8)	48(72.7)	0.619
≥60	26	8(22.2)	18(27.3)	
Gender				
male	84	27(75)	57(76.4)	0.15
female	18	9(25)	9(13.6)	
AFP				
≤20	31	11(30.6)	20(30.3)	0.517
>20	71	25(69.4)	46(69.7)	
HBV				
negative	19	10(27.8)	9(13.6)	0.067
positive	79	24(72.2)	55(76.4)	
Cirrhosis				
absent	27	11(30.6)	16(24.2)	0.49
present	75	25(69.4)	50(75.8)	
Intrahepatic metastasis				
absent	74	29(80.6)	45(68.2)	0.181
present	28	7(19.4)	21(31.8)	
Histological grade				
I, II	57	10(27.8)	47(71.2)	0.035*
III, IV	45	26(72.2)	19(29.8)	
Tumor size				
≤5cm	55	9(25.7)	46(69.7)	0.043*
>5cm	46	26(74.5)	20(30.3)	

P value represents the probability from a Chi-square test for different immunohistochemical scores of Ikaros in HCC tissues.

* , p<0.05.

We then tested relative molecular in 7 pairs of HCC and their matched adjacent liver tissue samples (Figure 6D). Western blot showed that phosphorylation of Erk1/2 and ETS1 was increased, and Ikaros expression decreased and CD133 expression increased in HCC tissue. Overall, we proposed that Ikaros interacting with CtBP played roles as transcription repressors in inhibiting CD133 expression via binding to P1 promoter, and MAPKs pathways indirectly regulated the expression of Ikaros and CD133 via ETS1 (Figure 6E).

## DISCUSSION

Although Ikaros plays an indispensable role in the development of leukemia, its role in solid tumors remains unknown. Yang *et al* examined the relationship between Ikaros expression and prognosis in 13 types of cancers and found that the relationship varied between cancers, even between the same cancers obtained from different databases [31]. In the current study, we confirmed for the first time that Ikaros plays an important role in HCC. We determined that Ikaros functions as a transcription repressor and inhibits CD133 expression in HCC.

In our study and other previous studies, CD133 was identified as a marker of CSCs in HCC [5, 6, 7]. Overexpression of CD133 in cancers is closely related to the poor prognosis associated [32]. Although several patterns of CD133 regulation have been reported, its mechanism still remains unclear. CD133 expression was regulated by Sp1/myc [33]. CD133 and CD133^+^ CSCs was found to be regulated by miR-142-3p in HCC [34], and Lupeol inhibited liver tumor-initiating cells and decreased CD133 expression through phosphatase and tensin homolog modulation [35]. CD133 expression is enhanced under hypoxic conditions via HIF-1α/mTOR signaling in glioblastoma [31, 36]. Furthermore, epigenetic modification was another important factor for the regulation of CD133 expression in many tumors. TGFβ has been shown to elevate CD133 expression via demethylation of the CD133 P1 promoter by inhibiting the expression of DNMT1 and DNMT3β [37] and Promoter methylation decreased CD133 expression in ovarian cancer and glioblastoma [33, 38].

In this study, we determined that Ikaros suppresses CD133 expression by directly binding to the CD133 P1 promoter. We found that Ikaros induced the differentiation of CD133^+^ cancer stem-like cells. Thus, we concluded that inhibition of the stemness property of CSCs was one functional role of Ikaros in HCC. Ikaros protein was partially localized to the cytoplasm of HCC samples and HCC cells (Figure 6A and Supplementary S5A and B). Wild-type Ikaros localized in the nucleus after forced Ikaros expression in HCC cells (Supplementary Figure S6A). This result indicated that the subcellular localization of Ikaros in HCC was important for its function. In addition, 157 phenylalanine, 162 arginine, and 184 arginine are necessary for Ikaros DNA binding activity and subcellular localization [26]. Mutant Ikaros could not localize to the nucleus and could not regulate CD133 expression when these three amino acids were mutated to alanine (Supplementary Figure S6B and C). However, part of the Ikaros was found to be subcellular localized in the cytoplasm of HCC as assessed by IHC. Altered localization of Ikaros may be difficult to act as a transcription factor in HCC cells, which requires confirmation in future studies.

In present study, we found that pETS1 was higher in HCC samples than their matched noncancerous liver specimens, and there is no difference in ETS1 expression. Although Ito et al reported that ETS1 was over-expressed in HCC [41]. Cowley et al reported that the p-ETS1 is the inhibitory form of ETS1 activity [30], therefore, we consider that phosphorylation modification is critical for ETS1 function and its activity in HCC samples. We also found that HCC samples Ito et al detected are HCC patients with hepatitis virus C (HCV), however, the HCC samples we used are HCC patients with hepatitis virus B (HBV). This different pathogens may result in the discrepancy of ETS1 expression between our data and that of Ito et al.

In this study, we confirmed that CtBP could interact with Ikaros as a complex, and repressed CD133 protein expression in HCC. CtBP, as a transcription repressor, plays important roles in multiples of cancers. CtBP1 interacted with Ikaros in pituitary tumor cells, and modulated their survival in response to hypoxia [42]. CtBP also by binding with p19Arf can inhibit the invasion of hepatocellular carcinoma cells [43] and decrease the sensitivity of breast cancer cells to mechanistically diverse cancer chemotherapeutic agents via p53-dependent and -independent roles [44]. In colon carcinomas, high levels of Zeb1 and CtBP were correlated with low levels of E-cadherin [45]. CtBP also drived the EMT, stem cell pathways and genome instability in breast cancer [46]. Usually, CtBP interacts with other transcription factors regulated the expression of down-stream genes. CtBP and PRDM16 co-control the brown and white fat-selective gene programs [47]. And in leukemic transformation of hematopoietic cells, Evi-1 interacted with CtBP [48] and repressed the transforming growth factor beta signaling [49]. Importantly, CtBP contributed to the activities of oncogenes Hdm2, Znf217, and multiple drug resistance genes MDR1 [50, 51, 52]. CtBP interacted with other transcription factors as complex, inhibited down-stream genes expression. In addition, CtBP might participate in multiples of biological process and interacted with other corepressors in HCC. In present study, Ikaros was one partner we determined in HCC. CtBP-Ikaros complex functioned as a transcription repression complex and inhibited CD133 expression. Other function of CtBP is needed to be explored in future.

In summary, we provide evidence that Ikaros acts as a transcription repressor in HCC, and our results indicate that Ikaros may be a potential target for anticancer therapy of HCC by enhancing its activity.

## MATERIALS AND METHODS

### Cell lines

Hep3B, PLC/PRF/5, and 293T cells were all obtained from the American Type Culture Collection (Manassas, VA, USA). Huh7 cells were obtained from the Riken Cell Bank (Tsukuba, Japan). SMMC-7721 cells were provided by the Cell Bank of the Institute of Biochemistry and Cell Biology, China Academy of Sciences (Shanghai, China). MHCC-LM3 cells were provided by the Liver Cancer Institute of Zhongshan Hospital, Fudan University (Shanghai, China). HCC-LY5 cells were established from a human primary HCC tissue in our lab.

### Cell culture

All cell lines used in this study were cultured in DMEM (Sigma-Aldrich) supplemented with 10% bovine serum (Hyclone), penicillin (500 U/ml), and streptomycin (500 μg/ml) and were incubated in 5% CO2 at 37°C. For the differentiation-inducing assay of CSCs *in vitro*, HCC cells were plated in six-well plates (NUNC, USA) in a monolayer culture with serum-free chemically defined medium (CDM). The CDM consisted of DMEDM/F12 supplemented with 0.5 × B27 supplements, 10 ng /ml basic fibroblast growth factor (bFGF) (Millipore), 10 ng epithelial growth factor (EGF) (Millipore), 500 U/ml penicillin, and 500 μg /ml streptomycin.

### Chromatin immunoprecipitation

Chromatin immunoprecipitation (ChIP) assay was performed in HCC-LY5, SMMC-7721, and MHCC-LM3 cells. The cells were cross-linked with 10% paraformaldehyde for 10 min at 37°C, and crosslinking was reversed with 1 M glycine for 5 min at 37°C. After washing with 1× PBS buffer, the cells were harvested in T-PER Tissue Protein Extraction Reagent (Thermo Scientific) for 5 min on ice and centrifuged at 2,000 *g* for 5 min. The precipitants were suspended in nuclei lysis buffer, and the DNA was shredded into fragments of 1,000 base pairs by sonication. Antibodies against Ikaros, CtBP and ETS1 (Santa Cruz Biotechnology) using protein G agarose beads (Sigma-Aldrich) were added and incubated overnight at 4°C. After reversing the crosslinks, the DNA was isolated and used for polymerase chain reaction (PCR) analysis. Primers for quantitative PCR (qPCR) are listed in Supplementary Table 1

### DNA pull-down assay

For DNA pull-down assays, 100 μg of nuclear protein per sample was incubated with 1 μg of biotinylated double-stranded oligonucleotides for 30 min at 37°C. Subsequently, DNA-protein complexes were incubated with streptavidin-agarose beads (Invitrogen) for 2 h at 37°C. After washing, the complexes were subjected to western blotting analyses. Sequences for DNA pull-down are provided as follows: Ikaros-normal:TCAGCTTTTGGGAATCTCCTGTCA; Ikaros-control: GC TTTTGGGAATCTCCTGTCA; Ikaros-normal: GAGAGTTTTTAGGAGGGTAGCTGC; Ikaros-mutant: GAGAGTTTTTAGTAGCTGC; ETS1-normal: CGAGTGAG CAACTTCAGGAATCATTGGAAGAAG; ETS1-mutant: CGAGTGAGCAACTTT TCCGCATCATTGTGAAGAAAG.

### Co-immunoprecipitation

Co-immunoprecipitation assays were performed using HCC-LY5, SMMC-7721, and MHCC-LM3 cells. After washing with 1× PBS buffer, the cells were harvested in T-PER Tissue Protein Extraction Reagent (Thermo Scientific) for 5 min on ice and were centrifuged at 2,000 *g* for 5 min. The precipitants were suspended in RIPA (Santa Cruz Biotechnology) lysis buffer for 50 min on ice and centrifuged at 5,000 *g* for 15 min. Antibodies against Ikaros and CtBP (Santa Cruz Biotechnology) with protein G agarose beads (Sigma-Aldrich) were added and incubated overnight at 4°C. After washing, the complexes were subjected to western blot analysis.

### Luciferase reporter assay

Cells were plated in 96-well culture plates for 24 h and were transfected with the appropriate constructs. Renilla and firefly luciferase activity were measured according to the manufacturer's instructions (Promega).

### Quantitative real-time PCR

RNA was extracted using TRIzol (Invitrogen) according to the manufacturer's protocol and was reverse-transcribed into cDNA using PrimeScriptTM RT Reagent Kit (TaKaRa). Primers for quantitative RT-PCR are provided in Supplementary Table 1.

### Western blotting

Western blotting was performed as described by the SuperSignal West Femto Maximum Sensitivity Substrate Kit (Pierce). The primary and HRP-conjugated secondary antibodies are listed in Supplementary Table 2.

### Plasmid constructs, lentivirus production, and cell transfection

Full-length human Ikaros (Origene) was subcloned into pWPXL (Addgene) at the BamH I and EcoR II sites. The CD133 full-length and deletion reporter constructs were generated and cloned into a pGL3-enhancer vector (Promega) at the Kpn I and Hind III sites. Three Ikaros single DNA binding sites mutants (K137A, R162A, and R184A) were generated using the megaprimer PCR method as previously described [39]. Virus packaging and cell transfection were performed as previously described [40]. Primers for cloning are provided in Supplementary Table 3.

### RNA interference-based gene knockdown experiments

Small-interfering RNA (siRNA) oligos targeting Ikaros, ETS1 and a negative control (Cat. No. B01001) were synthesized and annealed by GenePharma (Shanghai, China). Three fragments were designed to target the corresponding gene transcripts, and the silencing effects of the sequences were validated by western blotting analyses. Sequences for siRNA are provided as follows: siIkaros-1: AAGACCTGTGCAAGATAGGAT; siIkaros-2: GUCGUGGCCAGUAAUGUUATT; siIkaros-3: GACGCACUCCGUUGGUAAATT; siETS1-1: ACUUGCUACCAUCC CGUAC; siETS1-2: GGACAAGCCUG UCAUUCCU; siETS1-3: GCAGCAACUUG AAUUUGCUCACCAA.

### Magnetic-activated cell sorting

HCC-LY5, SMMC-7721, and MHCC-97L cells were magnetically isolated from CD133^+^ and CD133^−^ cells with corresponding antibodies using the Easysteo PE Selection Kit (StemCell Technologies) according to the manufacturer's instructions.

### Immunofluorescent confocal imaging

Cells were seeded onto glass slides for 24 h, fixed in 4% paraformaldehyde, and permeabilized with 0.5% Triton X-100 for 15 min. The slides were then incubated with primary antibody in blocking solution overnight at 4°C in a humidified chamber. The glass slides were then washed three times in PBS and incubated in Alexa 594-conjugated secondary antibody and 4′6-diamino-2-phenylindole (DAPI) in blocking solution for 40 min at 37°C in a humidified chamber. Images were obtained with a confocal laser microscope (IX-70, Olympus 40M, Olympus, London, UK).

### Immunohistochemistry

Paraffin-embedded tissue array sections (5μm in thickness) were prepared and immunodetections were detected by immunofluorescence according to the procedures described previously [6]. The results were visualized and photographed under an Axioskop 2 microscope (Carl Zeiss, Oberkochen, Germany) with a DP70 CCD system (Olympus, Tokyo, Japan).

### Plate colony formation assay

Two thousand cells were seeded into six-well culture plates and fixed seven days later using 10% formaldehyde for 30 min at 37°C. The cells were stained using GIEMSA (Sigma-Aldrich) for 30 min at 37°C. After washing, the cells cell colonies were quantified.

### MTT

Three thousand cells were seeded into 96-well culture plates and incubated for 24 h. Next, 100 μL MTT (5 mg /ml) was added to each well. Cells were incubated for 4 h at 37°C. The media was removed, and 100 μL DMSO was added in the dark for 2 h at 37°C. The OD was recorded at an absorbance of 570 nm.

### Fluorescence activated cell sorting

Huh7, PLC/PRF/5, HCC-LY5, and SMMC-7721 cells were incubated with a PE-conjugated CD133/1(AC133) antibody (Miltenyi Biotec, Germany) and sorted into CD133^+^ and CD133^−^ cell subpopulations on an Epics Altra flow cytometer (Beckman Coulter, USA)

### Drugs and drug-resistance test

Cisplatin (Sigma-Aldrich), Doxorubicin (Sigma-Aldrich), Sorafenib (Biochemparterner) were diluted to final concentrations with corresponding culture medium. After treated with drugs for 48 h, 100 μL MTT (5 mg /ml) was added. Cells were incubated for 4 h at 37°C. Removed media and added 100 μL DMSO in dark for 2 h at 37°C. OD was recorded absorbance at 570 nm.

### Tumor xenograft models

Six to eight-week-old BALB/c (*nu/nu*) mice were randomly divided into groups and inoculated with the suspended HCC cells (5×106 Huh7 cells, 5×103 CD133^+^ or CD133^−^ HCC cells were inoculated into the mice). At the end of the experimental period, the animals were sacrificed. Immediately after killing, xenograft tumors were weighed and fixed in neutral buffered formalin. Fixed tumor tissues were analyzed using western blotting analyses.

### Patients

One hundred and two human HCC tissue samples were obtained from patients who underwent surgical treatment at the First Affiliated Hospital of Zhejiang University (Hangzhou, China), the Qidong Liver Cancer Institute (Qidong, China) or the Guangxi Cancer Institute (Nanning, China). The 102 HCC patients included 84 males and 18 females (mean age: 52.21 years, ranging from 30 to 76 years). None of the patients received chemotherapy or other treatment before surgery. All procedures were performed under consensus agreements and in accordance with the China Ethical Review Committee. All tissue samples were fixed in 4% phosphate-buffered neutral formalin for at least 72 hr and routinely embedded in paraffin.

### Statistical analysis

Statistical analyses were performed using SPSS 13.0 software. The results were presented as the mean ± SD and compared using Student's *t* test. Survival analysis was performed using the Kaplan-Meier method. Statistical computations were performed using GraphPad Prism version 5.0. p<0.05 was considered significant. *p < 0.05; **p<0.01.

## SUPPLEMENTARY MATERIAL AND FIGURES


