# Supplementary material for: Inhibitory effects of transcription factor Ikaros on the expression of liver cancer stem cell marker CD133 in hepatocellular carcinoma
Source: Oncotarget. 2014 Sep 25;5(21):10621–35. doi: 10.18632/oncotarget.2524 (PMC4279398; doi:10.18632/oncotarget.2524)
Supplement: Supplementary file 1 [file oncotarget-05-10621-s001.pdf]

Inhibitory effects of transcription factor Ikaros on the expression of liver cancer stem cell marker CD133 in hepatocellular carcinoma

Supplementary Material

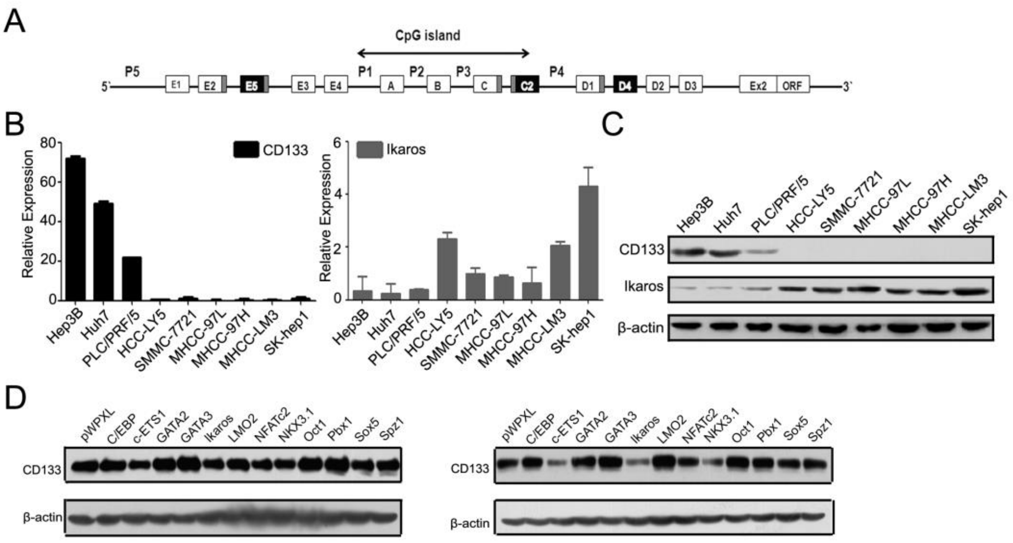

Supplementary Figure S1: P1 promoter was the key promoter of CD133 in HCC. (A) Five splicing patterns of the CD133 promoter. (B and C) q-PCR and immunoblot analysis of the expression of Ikaros and CD133 in nine HCC cell lines. (D) Predicted transcription factors regulated CD133 expression in Huh7 and PLC/PRF/5 cells.

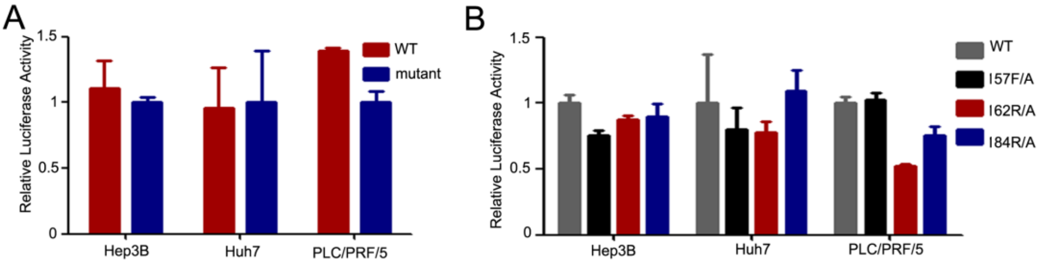

Supplementary Figure S2: Ikaros inhibited CD133 expression. (A) The promoter activity of mutant P1 after the transfection of Ikaros into Hep3B, Huh7, and PLC/PRF/5 cells. (B) The promoter activity of P1 after the transfection of mutant Ikaros (157F/A; 162R/A; 184R/A) into Hep3B, Huh7, and PLC/PRF/5 cells.

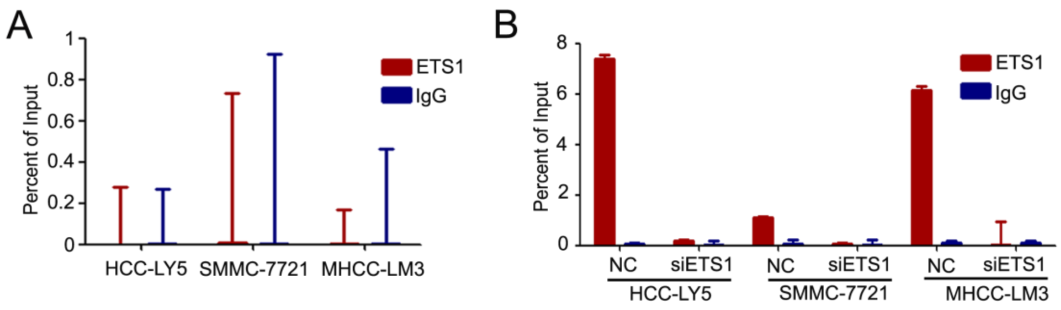

Supplementary Figure S3: ETS1 regulated the expression of Ikaros. (A) Binding of ETS1 on the CD133 gene promoter was assayed by chromatin immunoprecipitation. ChIP was performed using antibodies to ETS1 on HCC-LY5, SMMC-7721, and MHCC-LM3 cells. A negative control with irrelevant antibody (IgG) was included for comparison. PCR was performed on the input and bound fractions. (B) Binding of ETS1 on the Ikaros gene promoter was assayed by chromatin immunoprecipitation. ChIP was performed using antibodies to ETS1 on HCC-LY5, SMMC-7721, and MHCC-LM3 cells. A negative control with irrelevant antibody (IgG) was included for comparison. PCR was performed on the input and bound fractions.

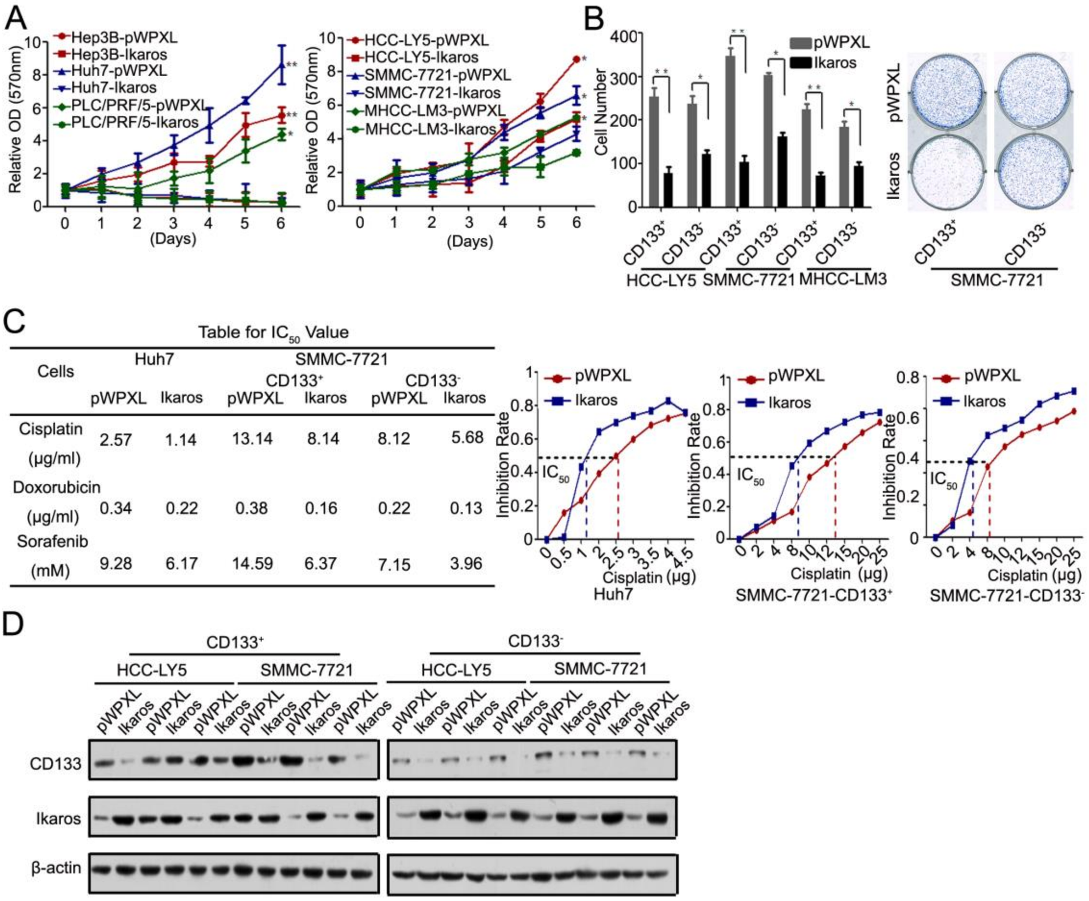

Table for IC_{50} Value

Cells	Huh7		SMMC-7721 CD133+		SMMC-7721 CD133-	
	pWPXL	Ikaros	pWPXL	Ikaros	pWPXL	Ikaros
Cisplatin (μg/ml)	2.57	1.14	13.14	8.14	8.12	5.68
Doxorubicin (μg/ml)	0.34	0.22	0.38	0.16	0.22	0.13
Sorafenib (mM)	9.28	6.17	14.59	6.37	7.15	3.96

Supplementary Figure S4: Ikaros repressed the activation of $CD133^+$ HCC cells. (A) MTT analysis of the tumor growth capacity of HCC cells when Ikaros was overexpressed. (B) Plate clone-formation assay analysis of the tumor growth capacity of $CD133^+$ HCC cells sorted from HCC-LY5, SMMC-7721, and MHCC-LM3 cells when Ikaros was overexpressed. (C) IC_{50} value for HCC cells treated with different drugs for 48 h. (D) Immunoblot analysis of Ikaros and CD133 expression in mouse tumor tissues. Data represent mean ± SD of three independent experiments with technical triplicates for each, and statistical analysis was performed using Student's *t* test. **, p<0.01; *, p<0.05.

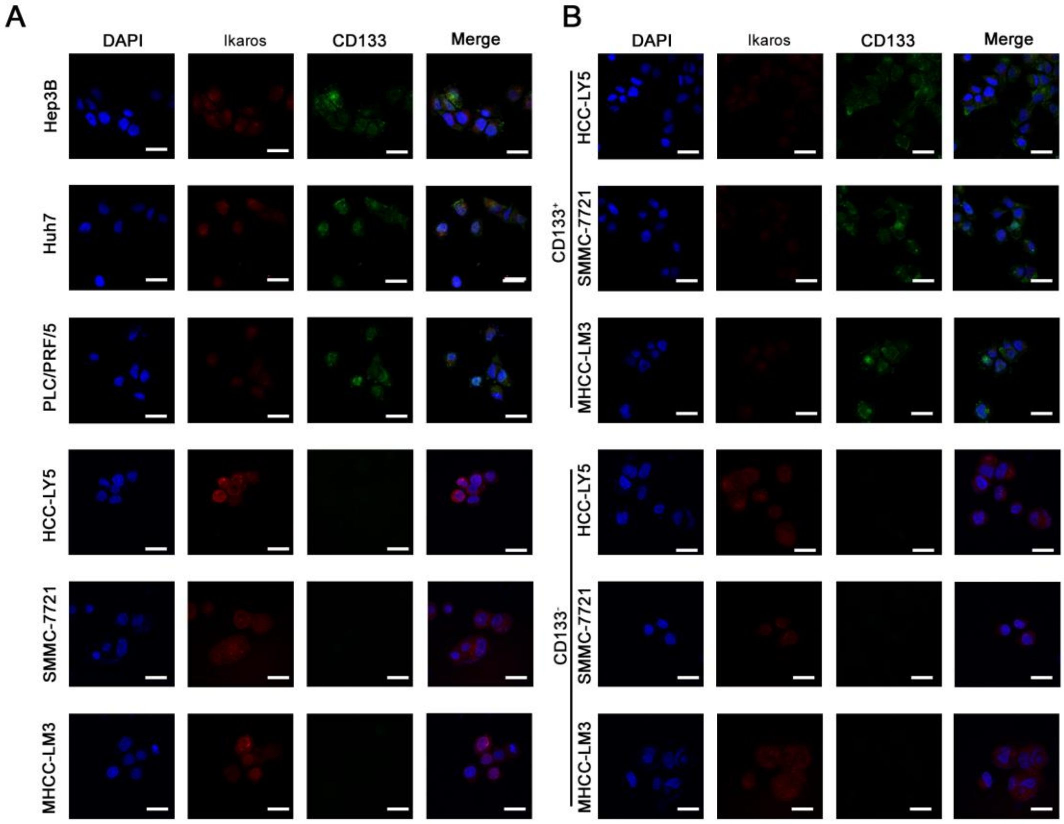

Supplementary Figure S5: The Ikaros partially localized to the cytoplasm of HCC cells. (A) Confocal imaging analysis of Ikaros co-localization in HCCs. Red indicates Ikaros; green indicates CD133; and blue indicates DAPI. Scale bar, 10 μm. (B) Confocal imaging analysis of Ikaros and CD133 colocalization in $CD133^+$ and $CD133^-$ HCCs. Red indicates Ikaros; green indicates CD133; and blue indicates DAPI. Scale bar, 10 μm.

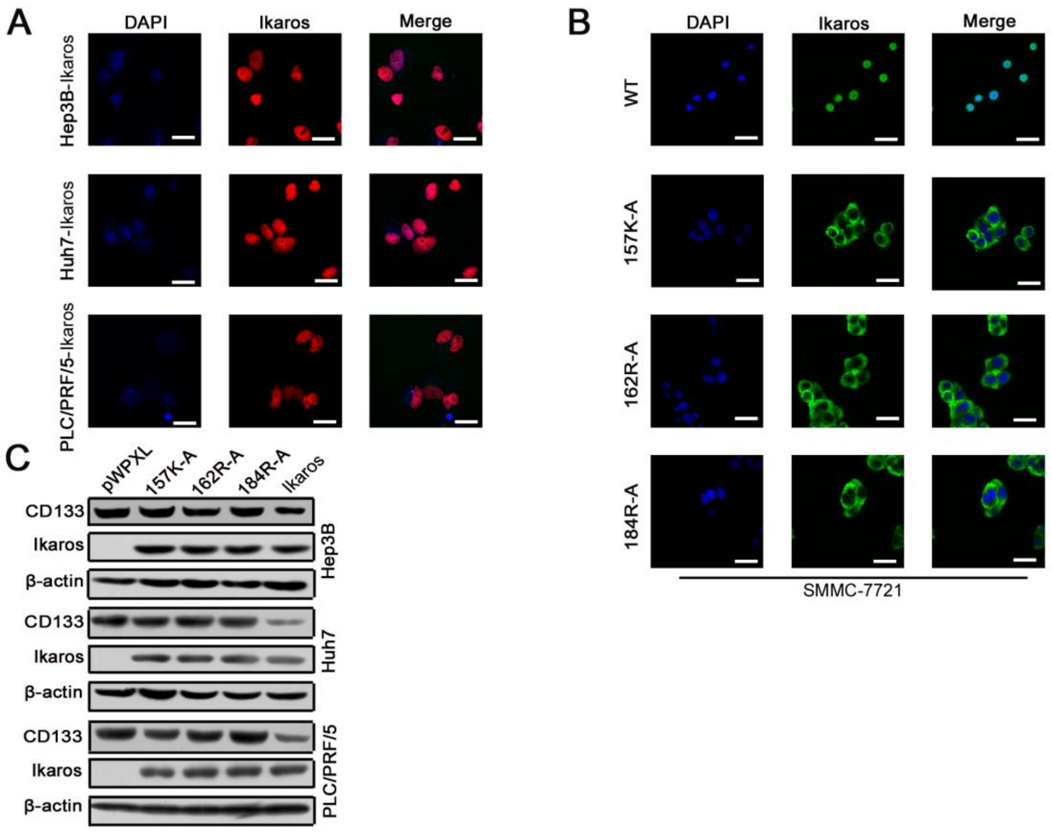

Supplementary Figure S6: Mutant Ikaros located in the cytoplasm of HCC cells. (A) Confocal imaging analysis of Ikaros when Ikaros was overexpressed in Hep3B, Huh7, and PLC/PRF/5 cells. Red indicates Ikaros, and blue indicates DAPI. Scale bar, 10 µm. (B) Confocal imaging analysis of mutant Ikaros when lysine 137, arginine 162, and arginine 184 of Ikaros were mutated to alanine. Green indicates Ikaros, and blue indicates DAPI. Scale bar, 10 µm. (C) Immunoblot analysis of CD133 expression when mutant Ikaros was overexpressing in Hep3B, Huh7, and PLC/PRF/5 cells.

Supplementary Table

Supplementary Table 1: Primer for qRT-PCR

Name	Primer Sequence
Ikaros-F	5`-TTGGTACTGCTGCACACTAA-3`
Ikaros -R	5`-TAGAGGTGCCTGCTCAACC-3`
ETS1-CD133-F	5`-GCGAAAGCCTGGTCTGAGC-3`
ETS1-CD133-R	5`-CGCAGTCACTTGTTAGAGTG-3`
ETS1- Ikaros -F	5`-CAGCAGGTGGCAACGCAAG-3`
ETS1- Ikaros –R	5`-CGCAGTCACTTGTTAGAGTG-3`
P1-F	5`-ACCTGGCCATGCTCTCAGCT-3
P1-R	5`-TCACGCGGCTGTACCACATAGA-3`
P2-F	5`-GTGACTAGGGCGGGAGCAG-3
P2-R	5`-TCACGCGGCTGTACCACATAGA-3`
P3-F	5`-GTGCCCGTCCAACCCAAACTT-3`
P3-R	5`-TCACGCGGCTGTACCACATAGA-3`
P4-F	5`-CGCCAAAAGCACTCCAGATGAC-3`
P4-R	5`-TCACGCGGCTGTACCACATAGA-3`
P5-F	5`-TGGATCTGGACCCCAGGAGTT-3`
P5-R	5`-TCACGCGGCTGTACCACATAGA-3`
Ikaros -F	5`-ATGGGCGTGCCTGTGAAATGA-3`
Ikaros -R	5`-GCCGTTCTCCAGTGTGGCTTCTT-3`
CtBP-F	5`-ACTGTGGCCTTCTGCGACG-3`
CtBP-R	5`-GGTGTGGTACATCAGGGCCC-3`
ETS1-F	5`-AGGCATTGTGGGTAATAACA-3`
ETS1-R	5`-AAGTCCACTGTCGCTGTCTC-3`

F, forward primer; R, reverse primer; RT, reverse-transcription primer

Supplementary Table 2: Antibodies used in this study

Antibody	Clone, host	Dilution	Company
For Western blotting			
Ikaros	H-100, rabbit polyclonal	1:200	Santa Cruz
CD133	W6B3C1, mouse IgG1	1:100	MACS
CtBP	E-12, mouse IgG1	1:500	Santa Cruz
p-ETS1	pT38, rabbit polyclonal	1:300	Invitrogen
ETS1	H-150, rabbit polyclonal	1:500	Santa Cruz
p-Erk1/2	Ser473, rabbit polyclonal	1:500	CST
Erk1/2	C67E7, rabbit mAb	1:500	CST
Cytokeratin 18	RGE53, mouse IgG1	1:2,000	Santa Cruz
Cytokeratin 19	A53-B/A2, mouse monoclonal IgG_{2a}	1:2,000	Santa Cruz
β-actin	AC-15, mouse mAb	1:20,000	Sigma
Secondary antibody	HRP conjugated goat anti-rabbit IgG	1:3,000	Sigma
Secondary antibody	HRP conjugated goat anti-mouse IgG1	1:3,000	Santa Cruz
For Immunohistochemistry			
CD133	W6B3C1, mouse IgG1	1:50	MACS
Ikaros	H-100, rabbit polyclonal	1:50	Santa Cruz
Secondary antibody	Envision kit (HRP, rabbit/mouse, DAB+)	Ready-to-use	DAKO
For Immunofluorescence staining			
Ikaros	H-100, rabbit polyclonal	1:50	Santa Cruz
CtBP	E-12, mouse IgG1	1:50	Santa Cruz
Secondary antibody	Alexa Fluor 594 anti-rabbit IgG (Cat No. A-21207)	1:100	Invitrogen
	Alexa Fluor 488 anti- mouse IgG (Cat No. A-21202)	1:100	Invitrogen

Supplementary Table 3: Cloned primer sequences

Name	Primer sequence
Universal R	5`-CTTGAAGCTTGTGGGGATCTGCCTCAGTCA-3`
1800-P1-F	5`-TGAGGTACCTTCAGTGCCTCTTTCAGT-3`
1181-P1-F	5`-TGAGGTACCGGAAGAAGGGAAAGCAAGCAC-3`
1020-P1-F	5`-TGAGGTACCCCACTAGGTCCCTCTCCCAG-3`
760-P1-F	5`-TGAGGTACCTTTGTAGCTTGTGCATCCATCC-3`
480-P1-F	5`-TGAGGTACCACAGAGCGGGAAGACCAATAG-3`
340-P1-F	5`-CTAGGTACCCTGCACACTAAGGATCCAAATG-3`
260-P1-F	5`-TGAGGTACCGGATTAGGCAACAGAAGGGTC-3`
150-P1-F	5`-TGAGGTACCTTGCAAGAAGGGAGTGCAGG-3`
70-P1-F	5`-TGAGGTACCGGCTACGTGGCGAAGCAGC-3`
Universal R	5`-ATTGAATTCTTAGCTCATGTGGAAGCGG-3`
Ikaros -157-F	5`-GCTTGATGTGTGCGAGCAGG-3`
Ikaros -162-F	5`-CAGGTTGCCTGCCTGGGG-3`
Ikaros -184-F	5`-GGCGTCCCTTGCGCGGCAG-3`
P1-mut-F	5`-CAGGAGAGTTTTTTAAAGGGTAGCTGCATT-3`
P1-mut-R	5`-CTTGAAGCTTGTGGGGATCTGCCTCAGTCA-3`
P2-F	5`-ACTGGTACCGGTCCAATCAGAGTGCGT-3`
P2-R	5`-ATG AAGCTTCCCTTAGCTCGCCAGA-3
P3-F	5`-ACTGGTACCGGCGGCAGCGGTGACTA-3`
P3-R	5`-ACTAAGCTTGCACACTCACCTCCGACT-3`
P4-F	5`-ACTGGTACCGACAGACCCCAGATAGCCT-3`
P4-R	5`-ACTAAGCTTAGCCTGGGTGACAGAGACT-3`
P5-F	5`-ACTGGTACCGCATGTCTGTCTCTGTGTCC-3`
P5-R	5`-ACTAAGCTTACTGACAACTGCCCACTGC-3`
IK-promoter-F	5`-AAGAGGTACCGATGCTGCGCTGGAATGAG-3`
IK-promoter-R	5`-AAGAAAGCTTCCAAGACTGATCCTCGGG-3`
ETS1-F	5`-ATGAACGCGTATGAAGGCGGCCGTCGATC-3`
ETS1-R	5`-TGGTCATATGTCACTCGTCGGCATCTGGCTT-3`
CtBP-F	5`-AAGGACGCGTATGGGCAGCTCGCACTTG-3`
CtBP-R	5`-AAGGGAATTCCTACAACTGGTCACTGGCG-3`
Ikaros -F	5`-ATTGGATCCATGGATGCTGATGAGGGTC-3`

Ikaros -R	5`-ATTGAATTCTTAGCTCATGTGGAAGC-3`
GATA2-F	5`-ATTACGCGTATGGAGGGGCGCCCGAG-3`
GATA2-R	5`-ATTGAATTCCTAGCCCATGGCGGTCAC-3`
GATA3-F	5`-ATTACGCGTATGGAGGTGACGGCGGAC-3`
GATA3-R	5`-ATTGAATTCCTAACCCATGGCGGTGACC-3`
LMO2-F	5`-ATTGGATCCATGTCCTCGGCCATCGAA-3`
LMO2-R	5`-ATTGAATTCCTATATCATCCCATTGATCTT-3`
NFATc2-F	5`-ATTGGATCCATGAACGCCCCCGAGCGG-3`
NFATc2-R	5`-ATTGAATTCTCATAATATGTTTTGTATCCAGC-3`
NKX3.1-F	5`-ATTGGATCCATGCTCAGGGTTCCGGAG-3`
NKX3.1-R	5`-ATTGAATTCTTACCAAAAAGCTGGGCTCC-3`
OCT1-F	5`-ATTGGATCCATGGCCCCACCGTGGATGAC-3`
OCT1-R	5`-ATTGAATTCTTAAGGTAAATCGTGTTTTCTT-3`
Pbx1-F	5`-ATTGGATCCATGGACGAGCAGCCCAGG-3`
Pbx1-R	5`-ATTGAATTCTCACTGTATCCTCCTGTCTG-3`
SOX5-F	5`-ATTGGATCCATGCTTACTGACCCTGATTTA-3`
SOX5-R	5`-ATTGAATTCTCAGTTGGCTTGTCCTGC-3`
Spz1-F	5`-ATTGGATCCATGGCCAGCTCTGCTAAGTC-3`
Spz1-R	5`-ATTGAATTCCTATCTTAGGCTGCTAGCTG-3`

F, forward primer; R, reverse primer; RT, reverse-transcription primer
